# Taste Perception and Water Swallow Screen Results in Old-Old Women

**DOI:** 10.3390/geriatrics3040083

**Published:** 2018-11-20

**Authors:** Rachel W. Mulheren, Ianessa A. Humbert, Anne R. Cappola, Linda P. Fried, Marlís González-Fernández

**Affiliations:** 1 Department of Psychological Sciences, Case Western Reserve University, Cleveland, OH 44106, USA; 2 Department of Physical Medicine and Rehabilitation, Johns Hopkins University School of Medicine, Baltimore, MD 21287, USA; mgonzal5@jhmi.edu; 3 Department of Neuroscience, Johns Hopkins University School of Medicine, Baltimore, MD 21287, USA; 4 Swallowing Systems Core, Department of Speech, Language and Hearing Sciences, University of Florida, Gainesville, FL 32612, USA; ihumbert@ufl.edu; 5 Rehabilitation Sciences, College of Health and Health Professions, University of Florida, Gainesville, FL 32612, USA; 6 Department of Neurology, University of Florida, Gainesville, FL 32612, USA; 7 Division of Endocrinology, Diabetes, and Metabolism, Perelman School of Medicine at the University of Pennsylvania, Philadelphia, PA 19104, USA; acappola@pennmedicine.upenn.edu; 8 Mailman School of Public Health, Columbia University, New York, NY 10032, USA; lf2296@cumc.columbia.edu

**Keywords:** swallowing, taste, water swallow screen, aging

## Abstract

Changes in both swallowing and taste commonly occur in advanced age, though the relationship between the two is unknown. This study examined the association between a water swallow screen test and taste identification and intensity rating. Participants included 47 community-dwelling women aged 85–94 years. Participants completed three trials of a water swallow screen and were observed for signs of aspiration, which, if present, indicated failure. Four pure taste stimuli at low and high concentrations and water were presented, and participants selected one of five taste labels and rated their intensity on the generalized Labeled Magnitude Scale. Ratios of intensity ratings were computed for each taste stimulus to compare the perception of low and high concentrations. The association between water swallow screen failure, correct taste identification, and taste intensity ratio was evaluated with logistic regression modeling, with mediating factors of frailty and number of comorbidities. Failure of three water swallow screen trials was associated with a higher taste intensity ratio for caffeine (bitter) and a lower taste intensity ratio for sucrose (sweet). Correct identification of taste, frailty, and number of comorbidities were not associated with failure of any number of water swallow screen trials. Intensity ratings of certain tastes may be associated with swallowing in old-old women. Heightened vigilance in this population may be necessary to prevent complications related to dietary intake.

## 1. Introduction

During swallowing, stimuli are transported from the mouth to the stomach via a system of integrated motor and sensory nerves under brainstem and cortical control. In addition to meeting the goals of nutrition, hydration and secretion management, timely and coordinated bolus transit prevents aspiration of foreign material into the airway. In the absence of medical compromise, research indicates that swallowing function is susceptible to age-related changes. Compared to younger adults, older adults evidence longer durations of swallowing events, greater amounts of oropharyngeal residue after swallowing, reduced opening of the upper esophageal sphincter, and reduced oropharyngeal pressures [1,2,3,4,5,6]. These changes may be related to sarcopenia [7,8] and sensory deterioration [9,10].

Age-related changes in swallowing function contribute to heightened morbidity and mortality. Changes in swallowing function were found to be significantly associated with malnutrition and respiratory infection in older adults living independently [11]. As a group, frail older adults evidenced more impaired swallowing than younger adults, such as a higher incidence of penetration and aspiration of food into the airway due to delayed closure of the laryngeal vestibule [12]; in the same study, frail participants who evidenced impaired airway protection also had higher 1-year rate of mortality. It is important for clinicians to screen for aspiration risk as well as to address oral hygiene in older adults who may be at heightened risk of aspiration, as poor oral hygiene is associated with aspiration pneumonia [13] as well as reduced taste perception [14].

The prevalence of self-reported changes in taste increases steadily with age [15], with corroboration from different methodologies of formal testing. Older adults may require higher concentrations of stimuli to detect and identify tastes, and may rate the same taste stimuli as less intense than younger adults [16,17,18,19]. Older individuals may perceive the same stimulus to fall into different taste quality categories (e.g., sweet, sour) than younger individuals, with less accurate identification of tastes [19,20]. One study indicated that older participants recognized tastes at higher thresholds than younger participants despite comparable sensitivity to a lingual touch test and capsaicin [17].

Research has examined the effect of taste on swallowing, with mixed results. Some studies have indicated that taste stimulation can impact swallowing parameters such as frequency [21,22], timing [23,24], oropharyngeal pressure [25,26,27] and amplitude of submental muscle contraction [28], and can improve airway protection during swallowing as measured by the Penetration Aspiration Scale [29]. Possible mechanisms for this effect include heightened cranial nerve input to the swallowing central pattern generators at the brainstem level [30,31] as well as enhanced activation of the cortical swallowing network [32]. In contrast, certain authors report no effect of taste on latency to swallowing initiation [33], swallowing apnea duration [34,35], or hyolaryngeal kinematics [36]. Based on these results, the effect of taste on swallowing is unclear.

It is possible that reduction in taste sensitivity may contribute to age-related changes in swallowing. One study reported that taste significantly reduced the onset time of submental and infrahyoid muscle activation (as measured by electromyography during swallowing) in younger but not older participants [37]. Submental muscle activation can serve as a marker of hyoid excursion, which indicates the onset of swallowing and co-occurs with the initiation of laryngeal vestibule closure for airway protection during swallowing. The finding that the earlier onset of activation with taste stimulation was not present in older adults could be explained by reduced taste sensitivity with aging, and the association between taste and swallowing physiology specific to airway protection is unknown.

Aspiration, or invasion of material into the airway, before, after, or during swallowing, can be probed by means of a water swallow screen. During such a screen, participants consume water and either pass or fail based on observed responses such as choking or coughing. A failed screen is indicative of positive aspiration risk, which has been substantiated by direct imaging of physiology and bolus flow [38,39,40]. A water swallow screen could be used as an initial step to determine whether taste sensitivity is related to risk for aspiration in older adults. The higher incidence of medical complications and age-related frailty may also contribute to changes in swallowing in older adults [41,42,43], and should be considered as a potential mediating factor in the association between altered taste sensitivity and swallowing.

The goal of this study was to document changes in taste perception in older individuals and to determine the association between taste perception and water swallow screen results to fuel future directions for research. We hypothesized that ratios of perceptual taste ratings would be associated with failure of at least one water swallow screen, such that participants who were less sensitive to discrimination between low and high concentrations (higher ratios of intensity ratings) for all tastes would be more likely to fail the screen. Second, we hypothesized that incorrect identification of tastes would be associated with failure of the water swallow screen. Finally, we predicted that frailty and the number of comorbidities would mediate these associations.

## 2. Materials and Methods

The study was approved by the Institutional Review Board. Participants were prospectively recruited from individuals enrolled in the longitudinal population-based Women’s Health and Aging Study II [44]. Inclusion criteria for the parent study at baseline in 1994 were women aged 70–79 who were among the two-thirds least disabled in the community in that age group with sufficient hearing for initial telephone screening, English proficiency, high level of physical function, intact cognition, and ability to attend onsite evaluation. Participants were invited to participate in the current study on the seventh visit of the parent study (14 years after initial enrollment). Additional criteria for inclusion in the sub-study were no reported history or diagnosis of dysphagia. Health information including number of comorbidities and frailty status (frail/non-frail) was extracted from the most recent data collection cycle of the parent study, such that all data for the sub-study (health information, swallowing, taste perception) were collected at the same time. All participants provided written informed consent.

The protocol for the water swallow screen has been previously published [43]. Based on previously developed methods [38,40], participants consumed 3 oz. of water by cup, with instructions to drink as they normally would without stopping. This task was repeated for a total of three trials, with at least 1 min between trials. Immediately after each trial, a trained research assistant observed participants for signs of aspiration, including changes in vocal quality or vocalization, stopping before the full amount was consumed, choking, coughing, and throat clearing for up to 1 min. Documentation of at least one of these behaviors resulted in a failed trial. Reliability was assessed by kappa statistics comparing a sample of live ratings by the research assistant and posthoc ratings of recorded trials by an investigator (as reported in Gonzalez-Fernandez et al. [43]).

Taste perception was assessed with four taste stimuli in weak and strong concentrations and distilled water. The following taste stimuli were presented in random order: caffeine (0.003 and 0.032 M) for bitter, sodium chloride (0.034 and 1 M) for salty, citric acid (0.002 and 0.128 M) for sour, and sucrose (0.15 and 1M) for sweet [45]. Perceived taste intensity was evaluated with the generalized labeled magnitude scale (gLMS) [46]. This scale consists of a vertical line ranging from 0 (“no sensation”) to 100 (“strongest imaginable sensation of any kind”), with possible ratings of intensity falling anywhere in between. Additional labels include 1.4 “barely detectable”, 6 “weak”, 17 “moderate”, 34.7 “strong”, and 52.5 “very strong”. The upper limit of the gLMS, “strongest imaginable sensation of any kind”, allows people with varying sensory perceptions to use the scale as it is not limited by previous experience but what is possible or imaginable. Participants were oriented to the scale, instructed to rate intensity rather than palatability of stimuli, and encouraged to utilize the entire length of the scale. To confirm understanding of the scale, participants had to demonstrate a logical increase in rating the loudness of a whisper, a conversation, and the loudest sound they had ever heard. If the participant did not understand how to use the scale on the first attempt, the practice rating with sounds was repeated once. The test was discontinued if the ratings of sound were not logical after the first two attempts. In order to assess taste identification for each stimulus, participants were asked to select one of five printed taste descriptors: sour, salty, bitter, sweet, neutral or no taste. Tastings were presented in cups numbered (1–9) per random order, and, after swallowing, participants rated intensity and selected taste identification immediately after each trial. Participants rinsed the oral cavity with water between trials.

Water swallow screen outcome was categorized as passed/failed based on the above methods. The accuracy of taste identification (by selection of one of five taste labels) for each stimulus was coded categorically (accurate/inaccurate) and tallied separately for the low and high concentrations (minimum 0, maximum 4). A ratio of these tallies was computed for low-to-high concentrations. Due to genetic differences, healthy individuals perceive the intensity of the same stimulus to be different (e.g., weaker or stronger). In order to account for these individual differences in taste perception, ratings on the gLMS were converted to ratios of low-to-high concentrations for each participant and within each taste category. In the parent study, frailty was categorized according to a validated phenotype [47,48] with classifications as frail (≥3), pre-frail (1–2), and non-frail (0). For the purposes of the current study, frailty was dichotomized into non-frail (0) and frail (1+) groups. The number of comorbidities was documented from participant reports of physician diagnoses.

Logistic regression modeling was used to determine the association of taste intensity and identification with water swallow screen outcome, as mediated by frailty and number of comorbidities. Outcome measures included frailty (frail/non-frail), number of comorbidities, ratios of low-to-high gLMS ratings for the four tastes, and taste identification (correct/incorrect) for the four tastes and water. All outcome measures were entered into three separate logistic regression models with water swallow screen outcome: (1) Failure of 1 trial; (2) Failure of 2 trials (3) Failure of all trials. Variables were selected for entry into the multivariate model according to cut-off of *p* < 0.25 as recommended by Bursac et al. [49]. Collinearity was assessed by Variance Inflation Factor (VIF) >10 and a tolerance value <0.1, and the Akaike Information Criterion method was used for model selection. Statistics were run on IBM SPSS 24 software (Armonk, NY, USA).

## 3. Results

### 3.1. Summary of Water Swallow Screen Results

Forty-seven females (85–94 years) participated in this portion of the study, and all completed three trials of the water swallow screen. Eight participants failed 1 trial, 10 failed two trials, and 16 failed all trials (36).

### 3.2. Taste Perception Results

**Figure 1:**
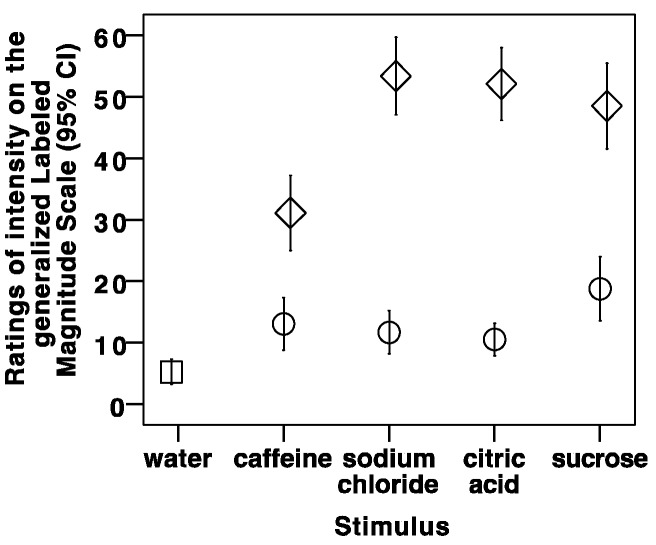
Ratings of intensity on the generalized Labeled Magnitude Scale for each stimulus, by low (circle) and high (diamond) concentration.

One participant did not participate in a trial of the sour stimulus, and another participant did not trial the salty and bitter stimuli. The taste data for these participants, specific to the taste stimuli that were not consumed, were not included in the analysis. Figure 1 summarizes the gLMS ratings by taste stimulus. As expected, the intensity of the neutral stimulus was rated lower than the four taste stimuli, and the low concentration of every taste stimulus was rated as less intense than the high concentration (Figure 1). The summary of low-to-high concentration ratios appears in Table 1. The minimum ratio of 0 for each taste indicates that at least one participant rated the intensity of the lowest concentration as 0 (“no sensation”). A lower ratio indicates more sensitive discrimination between the low and high concentrations for a specific taste whereas a higher ratio indicates less discrimination between low and high concentrations for a specific taste.

**Figure 2:**
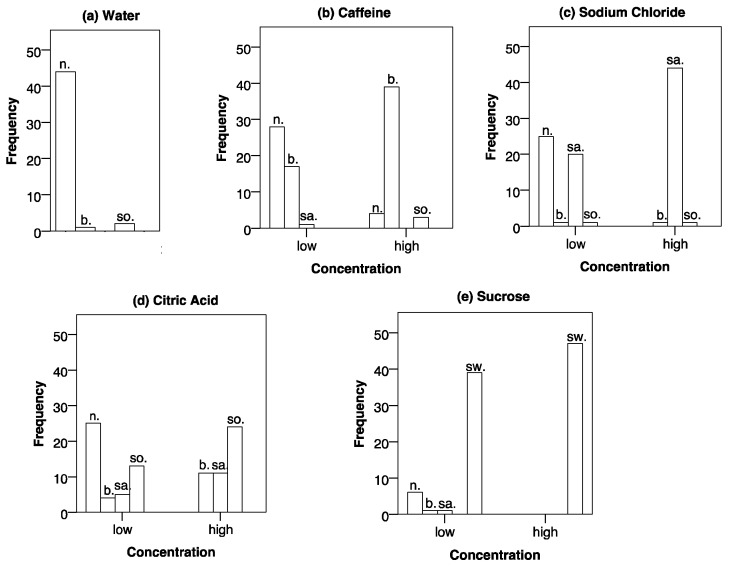
Taste identification frequency (y axis) for each stimulus by low and high concentration (x axis). Each plot represents a specific taste stimulus: (**a**) water; (**b**) caffeine; (**c**) sodium chloride; (**d**) citric acid; (**e**) sucrose. Bars are coded by reported taste quality: neutral (n), bitter (b), salty (sa), sour (so), sweet (sw).

Taste identification results are depicted in Figure 2. Distilled water was predominantly identified as neutral. At low concentrations, caffeine, sodium chloride, and sucrose were mostly perceived to be neutral; at the higher concentrations, the taste qualities of these taste stimuli were accurately identified by most participants. Perceptions of citric acid (sour) were more varied than other stimuli, with a correct identification frequency of less than 30 participants for both concentrations. The high concentration of sucrose (sweet) was the only stimulus that was identified by all participants without error.

Taste identification and intensity ratings were not significantly associated with failure of either one or two water swallow screen trials. Failure of all water swallow screen trials (total of three) was significantly associated with a higher caffeine gLMS ratio (*p* = 0.018, OR = 7.9, 95% CI: 1.42–44.08) and a lower sucrose gLMS ratio (*p* = 0.059, OR = 0.038, 95% CI: 0.01–1.13) in the same model (X^2^ (2) = 10.71, *p* = 0.005). No other measures were significantly associated with failure of all trials.

## 4. Discussion

This study investigated the association between water swallow screen outcome (pass/fail) and taste identification and intensity ratings in order to determine the effect of taste sensitivity on swallowing in older adults. A model including intensity rating ratios for bitter and sweet tastes was significantly associated with failure of all three water swallow screen trials, but not failure of one or two trials. Frailty and number of comorbidities did not modify these associations. These results suggest that sensitivity to discrimination between low and high concentrations of certain tastes may be associated with water swallow screen failure in old-old women. However, taste identification by labeling was not associated with water swallow screen outcome.

Different tastes may have nonuniform effects on swallowing due to differences in evolutionary purpose, method of transduction, and taste receptor types and number. A higher caffeine (bitter) gLMS ratio was associated with failure of three trials of the water swallow screen, indicating lower sensitivity to discriminating between low and high concentrations in participants who failed the three trials. Bitter taste is often rated as unpleasant and is thought to play a role in the avoidance of toxic substances [50,51,52]; therefore, participants may have felt resistance to consuming the bitter stimuli, which may have disrupted the swallowing response and yielded the association with water swallow screen results. In comparison to other tastes, bitter is transduced by many taste receptors [53,54]. In the same individuals who failed three water swallow screen trials, the sucrose (sweet) gLMS ratio was lower than in those who did not fail thee trials; sweet taste is pleasant and is generally accepted for consumption [52,55], thus it may have been more readily swallowed. These preliminary findings warrant further investigation with direct imaging of swallowing physiology.

In addition to providing sour and salty tastes via the facial, glossopharyngeal, and vagal nerves, the strong citric acid and sodium chloride stimuli may have induced a chemesthetic effect with additional stimulation of the trigeminal nerve [56,57]. Neither of these stimuli were associated with water swallow screen outcome in the present study, perhaps due to the robust response to taste coupled with chemesthesis that overcame any reduced taste sensitivity. Prior research found that a strong sour stimulus with presumed chemesthetic properties did not affect lingual movement during swallowing [58], but that it was associated with higher tongue pressures and submental muscle amplitudes during swallowing [25].

It is important to note that the stimuli in this study were pure tastes in order to exclude other sensory components (such as smell) that contribute to overall flavor perception. In addition to taste, nutritive foods and liquids vary by properties such as smell, texture, temperature, size, appearance, and sound during mastication. Olfaction may decline with aging in terms of smell identification and cortical activation in the olfactory neural network [59]. One study has suggested that olfactory stimulation can induce a more rapid initiation of swallowing in patients after stroke [60], though it is unknown whether age-associated changes in swallowing may be impacted by olfaction.

Taste and swallowing are both susceptible to between- and within-subject variability, as individuals may evidence slightly different perceptions of the same taste just as they may swallow the same bolus with different kinematics and timing in comparison to others and across trials [61,62]. Interestingly, there is evidence for increasing variability in taste sensitivity with age, suggesting different patterns of age-related change [62]. Higher variability was also reported in older participants in comparison to younger participants on measures of swallowing duration [63]. This variability may explain why the significant model was only present for three trials of the water swallow screen and not for one or two trials.

This study is limited in that we did not assess potential factors contributing to taste perception, such as genetic taster status, dentition, and current medications, which should be considered as mediators in future research. Genetic taster status categorizes individuals by the intensity with which they perceive a standardized concentration of phenylthiocarbamide or 6-n-propylthiouracil, yielding groups of non-, medium, and supertasters [64]. It is unknown whether water swallow screen outcomes may have varied by these groupings. The association between dentition and taste perception is unclear, and should be included as a factor in future studies. Although in some cases dentition has been found to have minimal to no effect on taste perception among older adults [65], other studies have found that natural or completely absent dentition may be associated with higher taste threshold (i.e., lower taste sensitivity) than full dentures in older adults [66,67]. Multiple medications can impact both taste perception and swallowing function [68,69], and should be considered in participant inclusion methods for future research.

The water swallow screen is not a full assessment of swallowing function, and future research with direct visualization of swallowing physiology and bolus transit is warranted. The current sample included female participants; due to differing degrees of age-related changes in taste sensitivity between males and females [16,70], different results may be obtained from a male sample in future studies. As age-related changes in taste that are localized to one portion of the oral cavity may be less apparent in whole-mouth stimulation tasks [20], future directions also include more precise measures of discrete taste function, with comparison to a younger sample.

## 5. Conclusions

Ratios of low-to-high ratings of taste intensity were associated with water swallow screen failure in old-old women, with a higher perceptual ratio for caffeine and a lower perceptual ratio for sucrose in those who failed three water swallow screen trials. Taste identification by labeling was not associated with water swallow screen outcome; further research is warranted to investigate the timing and kinematics of swallowing under direct imaging. Heightened vigilance in this population is necessary to prevent complications such as malnutrition and aspiration pneumonia.

## Figures and Tables

**Table 1:** Summary of low-to-high taste intensity (generalized Labeled Magnitude Scale) ratios.

	Caffeine	Sodium Chloride	Citric Acid	Sucrose
Median	0.36	0.17	0.18	0.31
Range	0–2	0–0.88	0–0.67	0–3.75
